# Late Onset Epstein Barr Virus Seropositive Posttransplant Lymphoproliferative Disorder in Two Renal Transplant Receivers

**DOI:** 10.4274/Tjh.2012.0017

**Published:** 2013-09-05

**Authors:** Saime Paydaş, Semra Paydaş, Mustafa Balal, Arbil Açıkalın, Melek Ergin, Emel Gürkan, Fikri Başlamışlı

**Affiliations:** 1 Çukurova University Faculty of Medicine, Department of Nephrology, Adana, Turkey; 2 Çukurova University Faculty of Medicine, Department of Oncology, Adana, Turkey; 3 Çukurova University Faculty of Medicine, Department of Hematology, Adana, Turkey; 4 Çukurova University Faculty of Medicine, Department of Pathology, Adana, Turkey

**Keywords:** Renal transplantation, Lymphoproliferative disorder, Burkitt lymphoma

## Abstract

Posttransplant malignancy is one of the most important complications of organ transplantation. Immunosuppressive drugs, viral infections such as human herpes virus 8 or Epstein-Barr virus, exposure to carcinogenic factors such as sun, and host factors can be etiologic factors in the development of malignant disease. In this paper we report 2 cases of late posttransplant lymphoproliferative disorder with malign behavior.

**Conflict of interest:**None declared.

## Abstract (tr)

Nakil sonrası dönemde malign hastalık gelişmesi organ naklinin en önemli komplikasyonlarından biridir. Malign hastalık gelişmesinde; immünsüpressif ilaçlar, human herpes virüs 8, Ebstein Barr virüs gibi viral enfeksiyonlar, güneşe maruz kalma ve kişiye ait özellikler etyolojik faktörler olarak sayılabilir. Burada nakil sonrası geç dönemde gelişen ve malign gidiş gösteren lenfoproliferatif hastalıklı iki olgu rapor edilmişti

## INTRODUCTION

After cardiovascular complications and infections, malignancy is the third most common cause of death in renal transplant receivers (RTRs) [1,2,3]. Malignant disorders account for the 20% of deaths in RTRs every year and this rate increases to 30% in cases followed for more than 20 years [4]. Increase in some cytokines such as transforming growth factor beta, interleukin-10 and vascular endothelial growth factor; immunodeficiency against viral infections; and DNA injury are etiologic factors in the development of posttransplant malignancy [5]. In transplant patients, Kaposi sarcoma, non-melanoma skin cancers, and non-Hodgkin lymphoma are the most common cancers, and the risk of these cancers has been found to be increased by 20-fold as compared with the normal population. The risk of renal cancer is reported to be increased 15-fold and a 5-fold increase risk has been found for melanoma, leukemia, and hepatobiliary, cervical, and vulvovaginal cancer. Two-to 3-fold increases have been reported for testicular, bladder, colorectal, lung, prostate, stomach, esophagus, pancreas, ovarian, and breast cancers [5].

## CASE REPORT

**Case 1**

**Figure 1:**
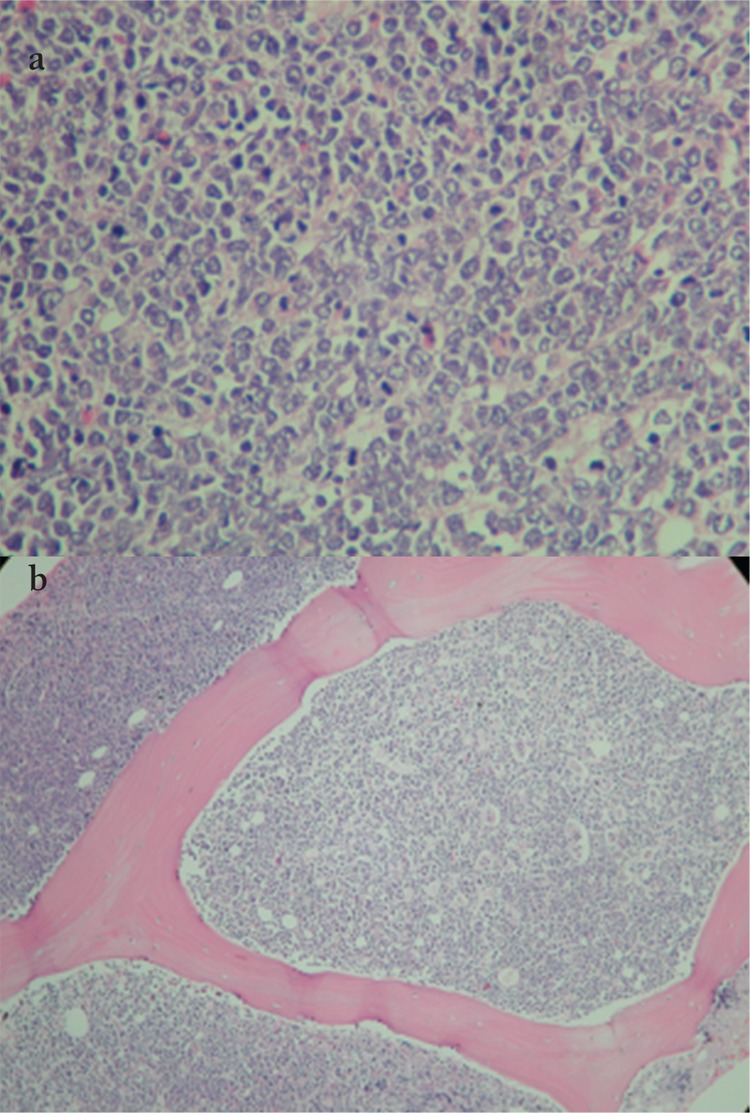
Diffuse infiltration of the bone marrow bymonotonous, medium-sized cells with multiple nucleoli,basophilic cytoplasm, and numerous mitoses. Starry skyappearance is present. Immunohistochemically, Pax-5 waspositive; Tdt, CD3, and MPO were negative in tumor cells.a) Burkitt lymphoma tumor cells with numerous mitoses(hematoxylin, 400×); b) monotonous, medium-sized cellsdiffusely infiltrating bone marrow with starry sky appearance(hematoxylin, 100×).

Renal transplantation after a short course of hemodialysis (HD) had been performed in a 22-year-old woman from her father in 1997. There was no severe complication in the early posttransplant period and she did not receive anti-thymocyte globulin (ATG) or high-dose corticosteroids. She had no hypertension and/or proteinuria and other complications. Drugs used in this case and the clinical outcome are summarized in Table 1. Calcineurin inhibitors (cyclosporin A [CysA] for 51 months and tacrolimus for 85 months), azathioprine at 100 mg/day, and corticosteroid at 4 mg daily for 4 years were used in the follow-up period. Corticosteroid administration was ceased when she was found to be positive for hepatitis C virus (HCV). At that time, her serum creatinine was 1.8 mg/dL and cyclosporine and azathioprine were substituted with tacrolimus and mycophenolate mofetil (MMF). In 2008 she wished to become pregnant and MMF was replaced by azathioprine. In the last year, she was receiving tacrolimus, and azathioprine. In December 2009, she was hospitalized due to fever, anemia, and thrombocytopenia. At this time, physical exam was negative except for the forearm cellulitis. Chest X-ray, abdominal ultrasonography and blood–urine cultures were found to be negative. Serologic tests for Salmonella, Brucella, human immunodeficiency virus, and cytomegalovirus (CMV) were found to be negative. Epstein-Barr virus (EBV) IgM was negative and EBV IgG was positive and HCV polymerase chain reaction was found to be positive. Piperacillin-tazobactam plus linezolid were prescribed but the fever continued. Bone marrow aspiration showed dysplastic changes and periodic acid Schiff (PAS)-negative blasts, which were compatible with acute lymphoblastic leukemia L3 or Burkitt cells. Histopathological bone marrow biopsy revealed post-transplant lymphoproliferative disease (PTLD), monomorphic PTLD, and Burkitt lymphoma (Figures 1a and 1b). There was diffuse infiltration of the bone marrow by monotonous, medium-sized cells with multiple nucleoli, basophilic cytoplasm, and numerous mitoses. Starry sky appearance was present. Immunohistochemically, Pax-5 was positive while terminal deoxynucleotidyl transferase (Tdt), CD3, and myeloperoxidase (MPO) were negative in tumor cells. Immunosuppressive drugs were ceased. Abdominopelvic magnetic resonance imaging (MRI) showed pelvic fluid and retroperitoneal lymph nodes. Unconsciousness developed and there was no evidence of nuchal rigidity, papillary stasis or lateralized neurologic findings. Cerebral MRI showed diffuse thickening and contrast uptake in the meningeal structures. Cytology of the lumbar puncture showed blastic infiltration. CODOX-M including cyclophosphamide, doxorubicin, vincristine, methotrexate, calcium folinate, granulocyte colony-stimulating factor, Ara-C and rituximab was prescribed, but uremia developed and HD was performed. Nevertheless, fever and hypotension developed and she died.

**Case 2 **

**Figure 2:**
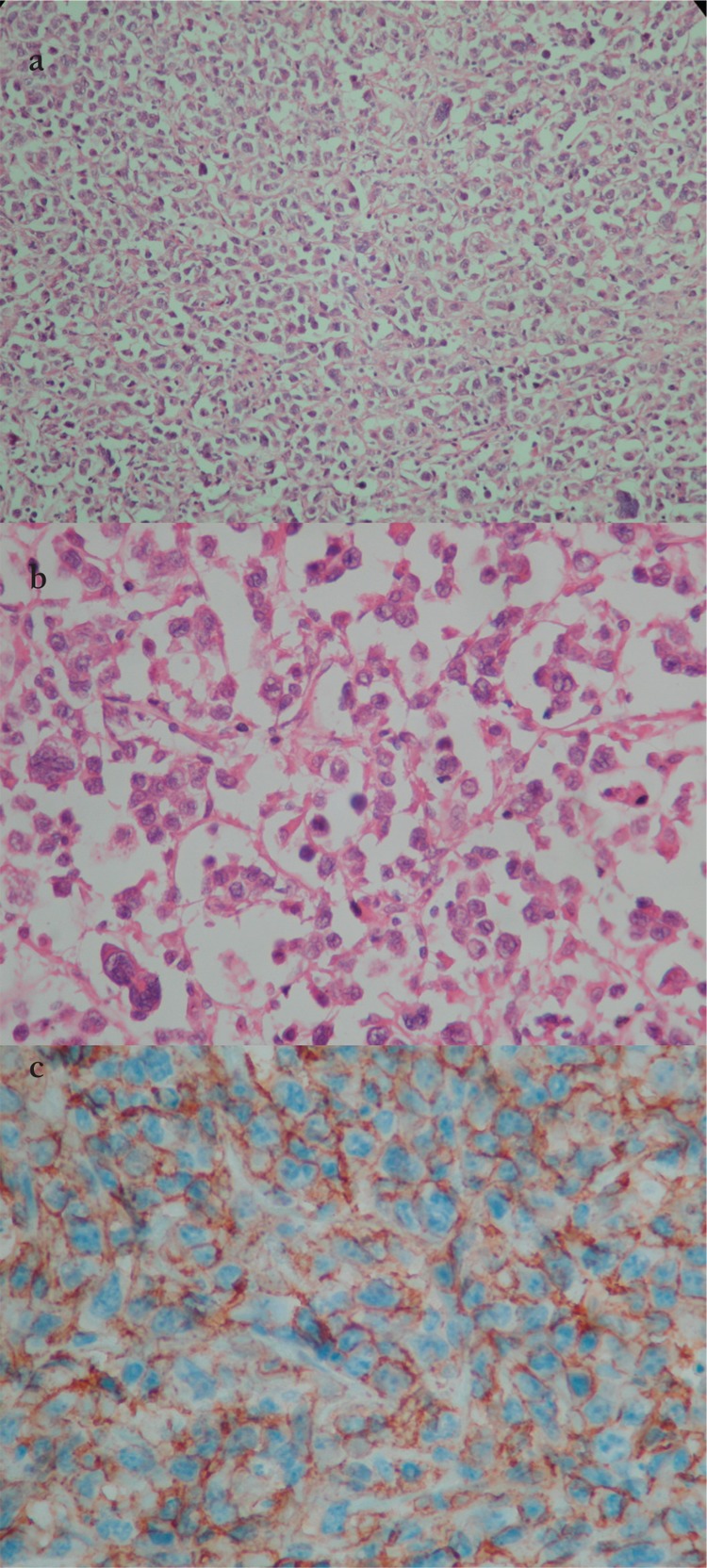
Diffuse large B cell lymphoma showingatypical large lymphoid cells with multilobated nuclei:a) hematoxylin–eosin, 100x; b) hematoxylin–eosin, 400x;c) tumor specimen CD20 positivity (immunohistochemistry400x).

A 17-year-old male was admitted to our unit with end-stage renal failure. He had history of atrophic kidneysince he was 7 years old. The patient received renal transplantation from his father after 2 years of HD in 1997. There was no severe complication in the early post-transplant period and neither ATG nor high-dose corticosteroid was used. During follow-up he received prednisolone and CysA plus azathioprine. At the end of 2 years, there was increase in blood urea nitrogen (BUN) and creatinine levels, and edema developed. Renal biopsy showed vascular rejection. Prednisolone was given 500 mg/day for 5 days, but renal function did not improve and HD was initiated again. The transplanted kidney was removed due to abscess formation 5 years after transplantation and renal biopsy showed chronic rejection. He developed pneumonia and imaging revealed ascites, cardiomegaly, pericardial effusion and pulmonary interstitial infiltrations. There was evidence of left ventricular hypertrophy, mitral annular calcification, and left atrial dilatation at echocardiography. Peritoneal biopsy showed active chronic inflammation and mesothelial cell hyperplasia. Upper endoscopy showed gastroesophageal reflux disease, hiatal hernia and Barrett’s metaplasia. In 2005 the patient was hospitalized due to abdominal pain, nausea and vomiting. Paraaortic multiple conglomerate lymph nodes and splenomegaly were detected upon abdominopelvic CT. Biopsy taken by laparotomy was compatible with non-Hodgkin lymphoma–diffuse large B cell lymphoma. Diffuse proliferation of large lymphoid cells with vesicular nuclei containing fine chromatin and nucleoli were found. Some tumor cells had multilobated nuclei. Immunohistochemically, leukocyte common antigen and CD20 were found to be positive while CD30 and CD3 were negative. EBV-encoded ribonucleic acid (EBER) was negative by in situ hybridization (ISH); see Figures 2a, 2b, and 2c. Six cycles of rituximab-doxorubicin-cyclophosphamide-vincristine-prednisone (R-CHOP) were administered. After chemotherapy, complete remission was achieved. In 2006, chemotherapy was completed. In the follow-up period, CT examination was negative for lymphoma (Table 2). In 2009, cadaveric renal transplantation was performed. Low-dose ATG (1 mg/kg daily) was given for 5 days and then maintenance sirolimus (target level: 3-12) and MMF (2 g daily) plus prednisolone were prescribed. In the last visit in 2010, BUN was 18 mg/dL, creatinine 0.7 mg/dL, hemoglobin 16.2 g/dL, hematocrit 49.2%, white blood cell count 7.7x10.9/L, and daily proteinuria 30 mg/day. EBV IgG was positive and IgM was negative.

## DISCUSSION

PTLD is 20-fold more common in patients receiving organ transplantation as compared with normal populations [5]. PTLD is related to viral infections, especially EBV [6]. EBV has a central role in the pathogenesis of PTLD [7,8,9], although not all PTLD is EBV-related. The most clearly defined risk factor for PTLD is primary EBV infection, which increases the risk for PTLD by 10- to 76-fold [10,11]. However, EBV positivity is not the rule. EBV-related viral disease and EBV-related malignant disease may develop with direct and indirect effects of the virus [12]. Fever, neutropenia, pneumonia, enteritis, meningitis, or encephalitis may develop secondary to the direct effects of the virus. Indirect effects of the immunomodulatory effect of the virus may cause increased risk of immune suppression and opportunistic infections via the secretion of cytokines, chemokines, or growth factors. Additionally, viral infections may change the surface antigen expression (for example, human leukocyte antigen) and provoke rejection reaction and/or contribute to oncogenesis with dysregulated cellular proliferation. Infection with one virus may stimulate the replication of other viruses (like CMV+HCV) or immunosuppression [13,14,15,16,17]. EBV positivity was present in both of our cases and HCV was present in one of them. The spectrum of disease ranges from benign polyclonal B cell infectious mononucleosis-like disease to malignant monoclonal lymphoma. The majority is of B cell origin, although T cell, NK-cell, and null cell tumors have been described. T cell PTLD has been demonstrated in 10% to 15% of cases, especially in the late transplant period; within allografts, it can be confused with graft rejection or other viral infections. Lymphomas comprise up to 15% of tumors among adult transplant recipients (51% in children), with mortality of up to 40% to 60%. Many deaths are associated with allograft failure after withdrawal of immune suppression during treatment of malignancy. Compared with lymphoma in the general population, PTLD has increased extranodal involvement, bad response to conventional therapies, and poorer outcomes. 

The use of muromonab-CD3 or antithymocyte globulin seems to be associated with an increased risk of PTLD, especially in the first year [18,19]. In RTRs belatacept was associated with an increased incidence of PTLD [20]. Tacrolimus is commonly associated with an increased risk of malignancy compared to cyclosporine [18,19]. mTOR inhibitors might protect against the development of PTLD.

PTLD includes a wide range of histopathology. Histopathological findings are important both for estimating the prognosis and treatment decision, and they have been classified in different ways. There are 4 groups [21]:

1) Early lesions: reactive and plasmacytic hyperplasia, infectious mononucleosis-like.

2) PTLD polymorphic: polyclonal (rare), monoclonal.

3) PTLD monomorphic: B cell lymphoma (diffuse large B cell lymphoma, Burkitt/Burkitt-like lymphoma), plasma cell myeloma, T cell lymphoma (peripheral T cell lymphoma and other types). 

4) Other types (rare): Hodgkin disease-like lesions (associated with methotrexate therapy), plasmacytoma-like lesions.

According to this classification, cases 1 and 2 were compatible with the third group.

The cessation or decreasing of the dose of immunosuppressive drug is efficient in two-thirds of cases of EBV-related PTLD. This possibility is low in cases with EBV-related conditions and develops in more than 1 year after transplantation. In these cases, there is a tendency for more malignant behavior. However, some cases may respond to the decreasing of immunosuppressive drug dosages, which should be considered in these cases [21]. 

In our first case, B cell lymphoma developed 12 years after transplantation and the patient presented with anemia and thrombocytopenia. Bone marrow aspiration/biopsy and cerebrospinal fluid were found to be positive for malignant cells. HCV and EBV IgG positivity were present in this case and there was no evidence of HCV-related liver disease. Two viral infections may be responsible as the risk factor for both the rejection and the development of malignant disease. In our second case, Burkitt lymphoma was localized in the abdomen and serum EBV IgG was found to be positive. Tumor specimen was negative for EBER by ISH. In the first case, ISH for EBER was not done due to the acid exposure of the bone marrow specimen.

 The case 1 of the current study patient both tacrolimus and CysA and the second case received only CysA. Additionally, our first patient was receiving immunosuppressives while the lymphoma developed, while in our second case lymphoma was detected 5 years after the cessation of immunosuppressive drugs. Azathioprine was used in both cases and MMF was used in the first case during the pre-lymphoma period. MMF was found to be relatively safe for development of PTLD in early and late periods [18,19].

In both of our cases, lymphoma developed in the post-transplant later period. For this reason, prognosis was poor and it was necessary to use anti-neoplastic drugs for the treatment of lymphoma. In the first case, lymphoma developed while the patient was receiving immunosuppressives, and in second case, PTLD developed 5 years after the cessation of immunosuppressive drugs. The first patient was treated with immunosuppressives for 12 years, but the second patient received them for almost 2 years. In case 1, chemotherapy was started, but the patient died with complications. Patient 2 was treated by R-CHOP. After 2 years, complete remission was achieved and a second renal transplantation was performed. Calcineurin inhibitor was not used, while mTOR+MMF was given without any adverse event. In general, the longer the cancer-free interval before transplantation, the smaller the recurrence risk. For most malignant neoplasms, a period of 2 to 5 years is recommended [22]. Informed consent was obtained.

In conclusion, although ATG is the main drug accused in the development of PTLD, both of our 2 patients had not been treated with ATG. Lymphoma develops generally while patients are receiving immunosuppressive drug(s) and regression of PTLD has been reported with the cessation of immunosuppressives. However, high-grade lymphoma developed in both of our patients and they were treated with aggressive combination chemotherapy. 

## CONFLICT OF INTEREST STATEMENT

The authors of this paper have no conflicts of interest, including specific financial interests, relationships, and/ or affiliations relevant to the subject matter or materials included. 

## Figures and Tables

**Table 1:**
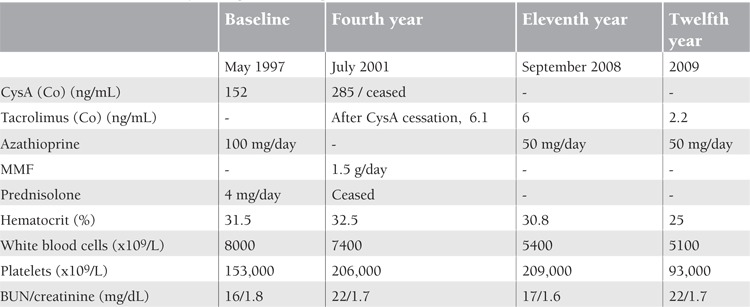
Clinical and laboratory findings of the first patient

**Table 2:**
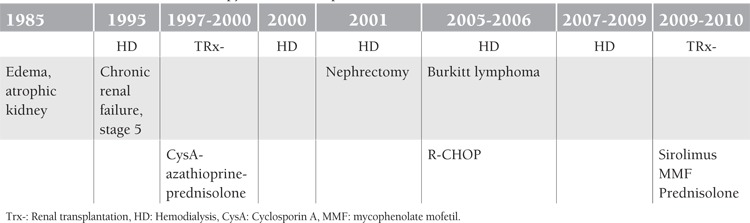
Clinical outcome and therapy of the second patient
